# Real-time tracking of the entangled pathways in the multichannel photodissociation of acetaldehyde†

**DOI:** 10.1039/d0sc00063a

**Published:** 2020-02-26

**Authors:** Chung-Hsin Yang, Surjendu Bhattacharyya, Lihong Liu, Wei-hai Fang, Kopin Liu

**Affiliations:** Institute of Atomic and Molecular Sciences (IAMS), Academia Sinica P. O. Box 23-166 Taipei Taiwan 10617 kliu@po.iams.sinica.edu.tw; Key Laboratory of Theoretical and Computational Photochemistry, Ministry of Education, Department of Chemistry, Beijing Normal University Beijing 100875 P. R. China fangwh@bnu.edu.cn; State Key Laboratory of Molecular Reaction Dynamics, Dalian Institute of Chemical Physics, CAS Dalian 116023 P. R. China; Aerosol Science Research Center, National Sun Yat-sen University Kaohsiung Taiwan 80424

## Abstract

The roaming mechanism, an unconventional reaction path, was discovered more than a decade ago in the studies of formaldehyde photodissociation, H_2_CO → H_2_ + CO. Since then, observations of roaming have been claimed in numerous photochemical processes. A closer examination of the presented data, however, revealed that evidence for roaming is not always unequivocal, and some of the conclusions could be misleading. We report here an in-depth, joint experimental and theoretical study of the title reaction. By tracking the time-evolution of the pair-correlated product state distributions, we decipher the competing, interwoven reaction pathways that lead to the radical (CH_3_ + HCO) and molecular (CH_4_ + CO) products. Possible roaming pathways are then elucidated and a more precise descriptor of the phenomenon is delineated.

## Abstract 2

Unraveling the entangled multi-channel dissociation pathways by a two-dimensional, time and product pair-correlation, measurement and *ab initio* calculations.

## Graphical abstract

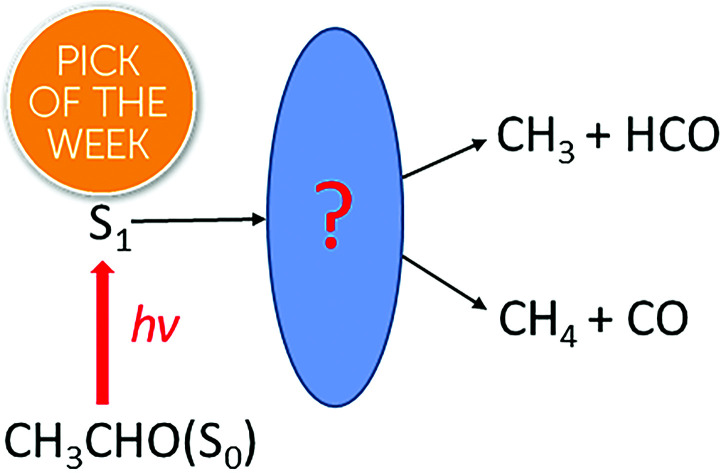

## 1. Introduction

The concept of the transition state (TS) is central to the understanding of chemical reactivity.^1^ An activated reaction is often envisioned to proceed from reactants to products along the minimum energy path (MEP) through a tight TS that serves as the bottleneck in breaking and forming bonds. Recent experimental and theoretical studies, however, documented a growing body of evidence that in some cases significant reactivity can also be derived from those trajectories bypassing the conventional saddle-point TS.^2–4^ A notable example, which sparked a surge of interest in non-TS dynamics, is the UV (ultraviolet) photodissociation of formaldehyde (H_2_CO).^5,6^ The formation of H_2_ + CO was experimentally observed and theoretically verified to proceed *via* two distinct pathways. In addition to the conventional TS mechanism that yields the product pair of a rotationally hot CO with a vibrationally cold H_2_, the so-called roaming mechanism leads instead to a highly vibrationally excited H_2_ together with a rotationally cold CO coproduct accompanied by a low total kinetic energy release (TKER). Theoretical analysis of the trajectory revealed that roaming arises from the incipient H⋯HCO radical channel where the two fragments do not have sufficient kinetic energy along the reaction coordinate to dissociate, but instead orbit each other (*i.e.*, roaming with significant kinetic energy tied to the centrifugal motion) and eventually undergo a radical recombination reaction by direct abstraction of the H atom from HCO at a long range to form H_2_ + CO, thereby emerging with distinct pair-correlated product distributions.

Since then, roaming has been claimed in numerous unimolecular dissociation processes,^7^ including the closely related acetaldehyde (CH_3_CHO) photodissociation.^8–14^ In those studies, experimental evidence for roaming is almost universally, with a few exceptions,^8,14^ based on the observation of a bimodal rotational state distribution (or sometimes a distribution with two Boltzmann rotational temperatures) for one of the molecular fragments. However, it is known that a bimodal rotational distribution of a reaction product can have several different mechanistic origins.^15^ Merely sighting a bimodal distribution, therefore, does not provide convincing evidence for roaming and such a claim is susceptible to controversy. For example, in the photodissociation of CH_3_CHO at 308 nm,^8–13^ the assigned roaming fraction varied from 15% of total CO yields in the initial report^8^ to a predominant 76–84% in more recent ones.^11,12^ A large disparity was also found at shorter wavelengths.^9,13,14,16^ Theoretical investigations so far did not help settle the dispute because of the complexity of the much higher dimensionality (15 degrees of freedom) and the involvement of nonadiabatic couplings of multiple potential energy surfaces (PESs).

The first absorption band of acetaldehyde (S_0_ → S_1_) spans from 340 nm to 230 nm and arises from excitation of an electron from the oxygen lone pair to the lowest π* orbital localized on the C–O bond. Upon excitation, several dissociation channels are possible, among which the CH_3_ + HCO and CH_4_ + CO channels dominate. Here, we report a joint experimental and theoretical study of the photodissociation of CH_3_CHO at 267 nm, focusing on these two channels,^17^ to clarify some of the confusing issues. Experimentally, a picosecond (ps) pump–probe approach (Fig. S1†) was employed to track the time evolution of product pair-correlated distributions^19^ (see the Methods section). With the aid of concurrent theoretical calculations [Methods], this set of two-dimensional – time and pair-correlation – results enable us to disentangle the multiple, interwoven dissociation pathways.

## 2. Two-dimensional perspective: time and pair-correlation

**Fig. 1:**
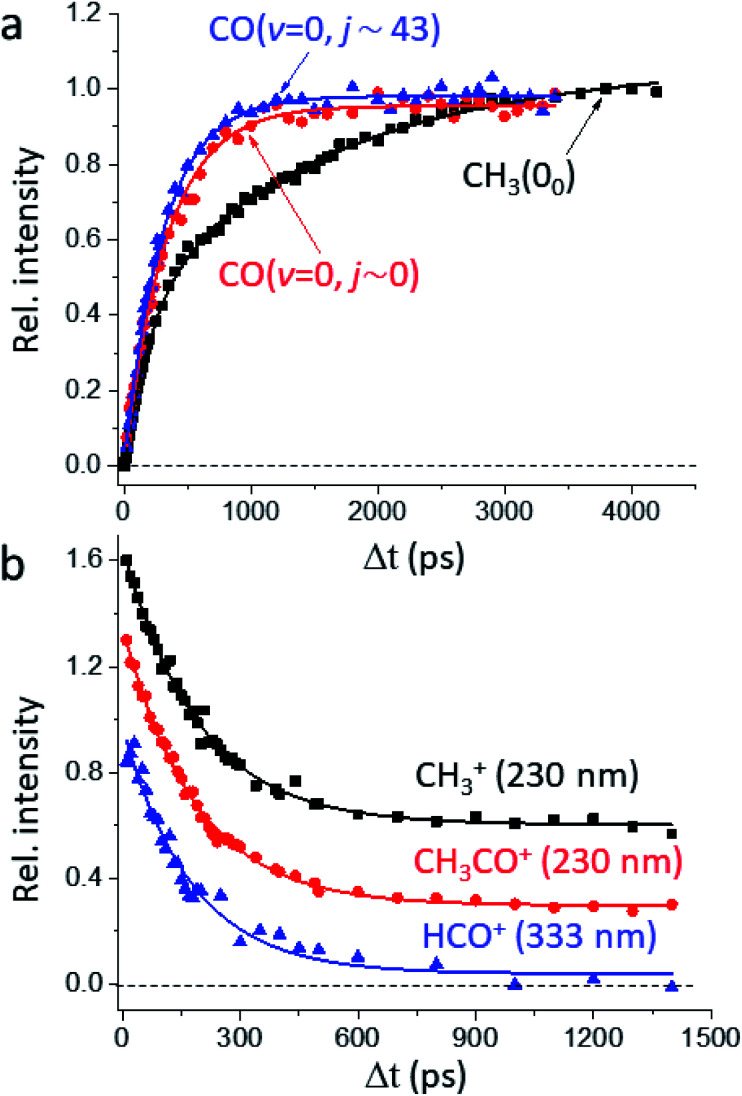
Temporal profiles of the probed species. (a) Depicted are the signals showing the growth of the REMPI-probed products with increasing pump–probe delay time (Δ*t*): CO(*v* = 0, *j* ∼ 0) in blue, CO(*v* = 0, *j* ∼ 43) in red, and CH_3_(0_0_) in black. While the rates of formation for low- and high-*j* states of CO are single-exponential and nearly identical (*τ* ∼ 340 ps and 310 ps, respectively), that for CH_3_ is distinct and clearly bi-exponential with *τ*_1_ ∼ 210 ps and *τ*_2_ ∼ 1650 ps. Note that the raw profiles (with the one-color backgrounds subtracted) actually show finite values at zero and negative time delays, which arise from the two-color dissociative ionization processes. Such backgrounds were accounted for with the aid of the non-resonant results shown in (b). (b) The temporal dependency of fragment-ions observed with the probe laser off-resonance from the respective REMPI bands: CH_3_^+^ and CH_3_CO^+^ at 230 nm, HCO^+^ at 333 nm. All profiles consistently display a single exponential decay of *τ*_d_ ∼ 190 ps. Three curves are vertically offset for clarity.

Fig. 1a presents the temporal profiles of the three products, CH_3_(0_0_), CO(*v* = 0, *j* ∼ 0), and CO(*v* = 0, *j*_pk_ ∼ 43), probed by the resonance-enhanced multiphoton ionization (REMPI) spectroscopic technique [Fig. S2†]. Both low- and high-*j* states of CO(*v* = 0) products display similar profiles, which can be fitted by the apparent, first-order kinetics with comparable time constants (*τ*) of 340 ps and 310 ps, respectively. On the other hand, the growth of CH_3_(0_0_) is clearly bi-exponential, strongly suggestive of complicated, multiple dissociation pathways. For the off-resonance probes (Fig. 1b), all time-dependent ion signals exhibit single-exponential decay with the same *τ*_d_ ∼ 190 ps. Those off-resonance ion signals were ascribed to the two-color, dissociative ionization processes from the pump-laser excited CH_3_CHO(S_1_). The observed profiles then correspond to the non-radiative decay of the S_1_ state to the T_1_ state by intersystem crossing (ISC) processes and/or to the ground S_0_ state *via* internal conversion (IC). The contaminations from those non-resonant backgrounds (Fig. 1b) were subtracted accordingly from the on-resonance data to unveil the profiles presented in Fig. 1a. For clarity, all formation *τ* herein refer to the overall time constant encompassing both this initial S_1_-decay (*τ*_d_) and the ensuing dissociation on the S_0_ or the T_1_ surfaces.

**Fig. 2:**
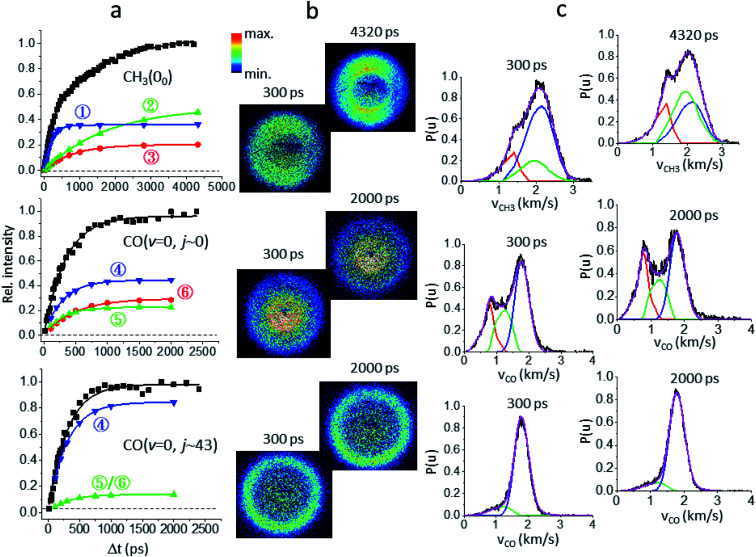
Unraveling the multiple components of each probed product. (a) The overall temporal profile (black, from Fig. 1a) of each product is decomposed into multiple components to recover the individual temporal evolution. The labeled numbers refer to the pathways listed in Table 1. All components exhibit single-exponential growths (shown by the lines) and the fitted time constants (*τ*) are given in Table 1. (b) For each species two representative background-corrected raw images, one at short time and the other at longer time, are exemplified. The observed (time-independent) anisotropic angular distribution of the CH_3_(0_0_) images arises from rotationally aligned fragments with respect to the recoil direction, in agreement with previous ns-experiments.^20^ (c) The resultant product speed distribution *P*(*u*; Δ*t*) of the partitioned component. The overall raw *P*(*u*; Δ*t*) is in black and the decomposed *P*(*u*; Δ*t*) is colored in accord with the corresponding temporal profile shown in (a).

Fig. 2 illustrates how to unravel the multiple reaction pathways for a given product from the time-of-flight (TOF) mass and the image data. Here, we define a pathway by its unique rate of formation and/or the distinct product pair-correlated distribution. Exemplified in Fig. 2b are two raw difference-images (with the pump- and probe-only backgrounds subtracted from the image with both lasers on) for each of the three products. The resultant product speed distribution *P*(*u*; Δ*t*) and the partitioned components are shown in Fig. 2c [see Fig. S3–S5† for the complete sets of time-resolved *P*(*u*; Δ*t*)*s*]. Combining each partitioned *P*(*u*; Δ*t*)-component (or path) with the overall TOF profile (Fig. 1a) then gives the individual temporal profile, as presented in Fig. 2a [see the Methods section for details].

## 3. Tracking the CH_3_ + HCO radical channel

**Fig. 3:**
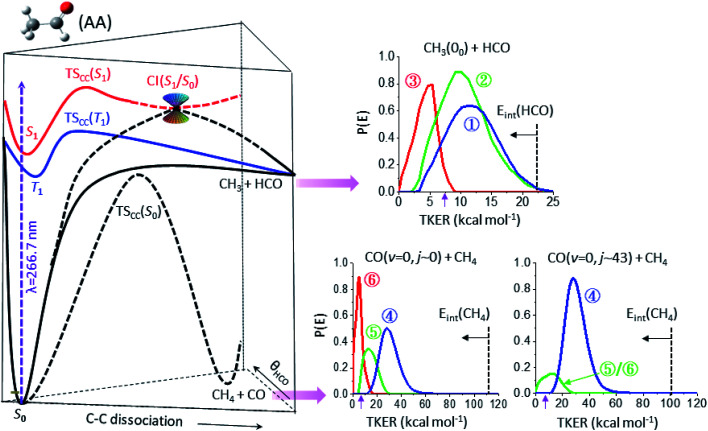
The final product kinetic energy distributions and the potential energy surfaces that lead to their formations. (Left) Schematic of three potential energy surfaces of CH_3_CHO(AA), S_0_ (black), T_1_ (blue), and S_1_ (red), showing the correlation with the CH_3_ + HCO and CH_4_ + CO fragments. (Right) The final kinetic energy distributions (Δ*t* → ∞) of the individual components of three different product channels: top for CH_3_(0_0_) + HCO, lower-left for CO(*v* = 0, *j* ∼ 0) + CH_4_, and lower-right for CO(*v* = 0, *j* ∼ 43) + CH_4_. The color code is the same as that in Fig. 2c and the vertical dashed lines mark the energetic limits. By conservation of energy, the *P*(*E*) distribution can be regarded as a mirror image of the internal energy distribution of the co-fragments with the horizontal arrow indicating the increase of internal energy. The purple arrow near 7.4 kcal mol^−1^ marks the energetic limit for triple fragmentation.

Fig. 3 summarizes the partitioned TKER distributions, *P*(*E*), of the final products (Δ*t* → ∞), along with the schematic of PESs. The overall distribution of CH_3_(0_0_) + HCO (black in Fig. 2c) displays a clear bimodal structure in accord with previous ns-laser studies, reflecting the internal energy distribution of the HCO coproduct. Significantly, adding the temporal evolution information unveils three distinct pathways. This product channel adiabatically correlates with both T_1_ and S_0_ surfaces. All three distributions peak substantially away from zero TKER, suggesting dissociations over exit-channel barriers and thus ruling out the barrierless pathway from the S_0_ equilibrium minimum.

The low energy component (red) accounts for about 19% of the branching fraction and has a long *τ* of 800 ps. The blue and green components are translationally hotter and exhibit remarkably similar *P*(*E*)-distributions. What differentiates the last two is the vastly different rates of formation. The blue path (36%) is fast and its formation time (190 ps) is essentially the time for S_1_-decay, indicating that once the S_1_ state is deactivated, CH_3_ + HCO is produced within a few ps. In marked contrast, the green pathway (45%) proceeds substantially slower (*τ* ∼ 1.8 ns), signifying possible isomerization during the course of dissociation (*vide infra*). Intriguingly, it ends up with a similar energy disposal to the fast-forming blue-component.

## 4. Tracking the CH_4_ + CO molecular channel

As for the CH_4_ + CO channel, three distinct paths are identified for the low-*j* states of CO(*v* = 0). The intense red-path (30%) with *τ* ∼ 480 ps yields low kinetic energy, *f*_TKER_ ∼ 0.05. By virtue of energy conservation, the CH_4_ coproducts must be highly internally excited. As is seen, a large fraction of the red component falls within the energy range for triple fragmentations to H + CO + CH_3_. This possibility, however, can be discarded from two previous reports.^21^ Therefore, we conclude that those highly excited CH_4_ co-fragments must be metastable to secondary dissociation, at least up to 40–70 ns of the pump–probe delay of the previous ns-laser experiments.^18,22^

The remaining two pathways, blue (46%) and green (24%), have similar *τ* ∼ 300 ps, but differ in TKER with *f*_TKER_ ∼ 0.28 and 0.13, respectively. Hence, the corresponding CH_4_ coproducts from these two paths are also highly excited. Taken together, all three paths yield internally hot CH_4_ coproducts with energy content far exceeding half of the C–H bond dissociation energy (Fig. 3).

When imaging the high-*j* states of CO(*v* = 0), only two features are notable and both proceed at about the same rates, *τ* ∼ 300 ps. The dominant blue feature (86%) is nearly the same – both in rate and TKER – as the blue component for *j* ∼ 0. The distributions of both *j*_CO_ states peak at the same recoil energy of 28.6 kcal mol^−1^; then, by conservation of energy, the difference in the probed CO rotational energy is entirely compensated for by the corresponding CH_4_ internal energy. A similar behavior was reported in a previous study at 248 nm.^14^ The weaker, lower-energy feature (green) encompasses an energy range over the green and red components for *j* ∼ 0. The peak profile and the overall *τ* of 310 ps suggest that its main contribution proceeds *via* the same path as that of the green component for *j* ∼ 0. Remarkably, a casual inspection of the *P*(*E*) distribution of either low or high *j*_CO_ indicates that none of the CH_4_ coproducts are born with internal energy less than ∼50 kcal mol^−1^, in sharp contrast to the result deduced from the IR emission of CH_4_ products photolyzed at 308 nm (ref. 10 and 12) or 248 nm.^16^

## 5. Uncovering the dissociation pathways

### 5.1 CH_3_ + HCO channel

Table 1 summarizes the characteristics of the partitioned components of the three products. To unravel their reaction pathways, we first noted that a previous calculation^23^ predicted a conical intersection (CI) between S_1_ and S_0_ at a fairly elongated C–C bond configuration, which provides a facile pathway to dissociation. This CI is preceded by a barrier, TS_cc_(S_1_), located at ∼103 kcal mol^−1^ above S_0_;^23^ an additional image of CH_3_ was then acquired with photolysis at 286 nm (photon energy of ∼100 kcal mol^−1^). As evidenced in Fig. S6,† the resultant *P*(*E*) distribution features a single Gaussian-like peak near 11 kcal mol^−1^ and the lower-energy structure observed at 267 nm is entirely absent. Hence, the red feature (near 5 kcal mol^−1^) of the radical channel in Fig. 3 is assigned to the CI(S_1_/S_0_) pathway.

**Table 1:** Characteristics of the multiple components retrieved from the time-resolved pair-correlation data and the assigned photodissociation pathways leading to the CH_3_ + HCO and CH_4_ + CO channels

Probed product	Component^a^	*τ* (ps)^b^	Branching^c^	〈*f*_TKER_〉	Assigned pathway
CH_3_(0_0_)	Blue	190 ± 10	36%	0.51	① TS_cc_(T_1_)
	Green	1750 ± 150	45%	0.45	② Isom.(S_0_, T_1_)
	Red	800 ± 50	19%	0.18	③ CI(S_1_/S_0_)
CO(*ν* = 0, *j* ∼ 0)	Blue	300 ± 15	46%	0.28	④ TS_cc_(S_0_)
	Green	290 ± 15	24%	0.13	⑤ Non-TS_cc_(S_0_)
	Red	480 ± 30	30%	0.05	⑥ CI(S_1_/S_0_)
CO(*ν* = 0, *j* ∼ 43)	Blue	300 ± 20	86%	0.31	④ TS_cc_(S_0_)
	Green	310 ± 20	14%	0.12	⑤/⑥ Non-TS_cc_(S_0_)

a The color-codes correspond to those depicted in Fig. 2–4.

b The growth of each partitioned component can be fitted by an apparent, first-order kinetics of B(1 − exp(−*t*/*τ*)). The quoted error represents ± (one standard deviation) from the fitting.

c The sum of branching fractions for a given product is set to unity. The typical error of each entry is ±2%.

This pathway is mediated by the gradient difference vector at the CI and asymptotically correlates with CH_3_(^2^A_2_′′) + HCO*(^2^A′′). At 267 nm, however, this electronically excited product pair is energetically inaccessible. Recalling that CI(S_1_/S_0_) occurs at a large C–C distance, the observed CH_3_(^2^A_2_′′) + HCO(^2^A′) ground-state pair is therefore ascribed to the result of inter-fragment quenching of the excited radical pair – a non-reactive event akin to the roaming reaction. The long formation time of 800 ps may reflect the additional time needed for this roaming-mediated quenching process.

The blue component in the CH_3_ + HCO channel is produced at about the same rate as that of S_1_-decay, which can readily be ascribed to a rapid, direct dissociation on T_1_ after ISC. This pathway must surmount a late exit-barrier of TS_cc_(T_1_), thereby causing a substantial TKER; the observed *f*_TKER_ ∼ 0.51 supports this assignment. The green component, on the other hand, takes an order-of-magnitude longer to form, yet with a very similar energy release (*f*_TKER_ ∼ 0.45) to that of the blue one.

Previous studies indicated that the ISC of S_1_ → T_1_ is a facile process and competes favorably with the direct IC in the Frank–Condon region, with the preference increasing with increasing photolysis energy.^13,18,24–27^ Thus, the dominant pathway for relaxing S_1_ to S_0_ at 267 nm is most likely a cascade of ISC pathways of S_1_ → T_1_ → S_0_ in the acetaldehyde (AA) configuration space. A number of feasible isomerization pathways on the S_0_ surface have recently been identified near the T_1_-dissociation threshold.^28^ Present theoretical calculations further suggest that the isomerization of vibrationally hot acetaldehyde to vinyl alcohol (CH_2_CHOH, VA) could become even more competitive at higher energies. More importantly, the S_0_ and T_1_ states are nearly degenerate in the structural landscape of the T_1_ minimum of VA – from an internal rotation of the terminal CH_2_ group, thus facilitating the S_0_ → T_1_ transition, (ISC)_VA_. Once on the (T_1_)_VA_ surface, a facile enol–keto isomerization can occur after surpassing a barrier TS(T_1_)_VA_ located at 105 kcal mol^−1^, taking the system back to the AA configuration, (T_1_)_AA_. Apparently, the last step must take place before reaching TS_cc_(T_1_) so that the ensuing dissociation will leave a similar dynamical imprint to that of the direct T_1_-dissociation pathway of the blue component. This complex, winding interconversion – from (AA → VA)_S_0__ to (T_1_)_VA_*via* (ISC)_VA_, followed by (VA → AA)_T_1__ – over the S_0_ and T_1_ surfaces delays the formation of CH_3_ + HCO (green).^29^ It should be noted that these isomerization and ISC processes are likely reversible, meaning that they could undergo multiple interconversions rather than a single step, thus further delaying the formation of final products. For clarity, Fig. 4 depicts the three pathways that lead to the radical channel, as well as those to the molecular channel as discussed below.

**Fig. 4:**
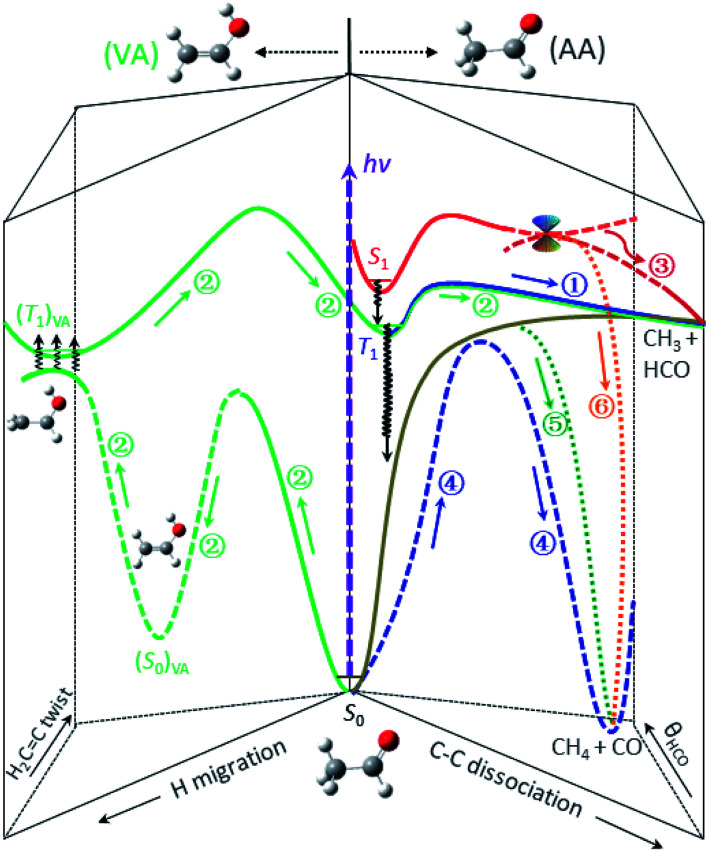
The roadmap of CH_3_CHO photodissociation at 267 nm (*hv* = 107 kcal mol^−1^). Six distinct, color-coded pathways are identified and elucidated for the formation of the CH_3_ + HCO and CH_4_ + CO channels. The labeled numbers refer to those in Table 1 and the black wavy arrows denote the ISC transitions. For clarity, detailed characterization of the key stationary points and the relevant energetics is presented in Fig. S8.†

### 5.2 CH_4_ + CO channel

As for the CO + CH_4_ molecular channel, the lowest energy feature (red in Fig. 3) is ascribed to the CI(S_1_/S_0_) path – similar to the radical case. This bifurcated CI-path is now governed by the derivative coupling vector at the S_1_/S_0_ intersection point and yields either a hot CH_3_CHO(S_0_) or the ground-state pair of CH_3_ and HCO. Either way, the subsequent formation of CO + CH_4_ could be attributed to the roaming reaction. Its *P*(*E*) distribution peaks around 5.7 kcal mol^−1^, similar to that of the red component (also *via* the CI(S_1_/S_0_)-path) in the radical channel.

The molecular products CH_4_ + CO are exclusively formed on the S_0_ surface. Three possible ways can deactivate S_1_ at 267 nm: IC of S_1_ → S_0_, CI(S_1_/S_0_), and ISC of S_1_ → T_1_. The red component has been assigned above to the CI(S_1_/S_0_) path. As mentioned earlier, the relaxation of S_1_ to S_0_ is dominated by a cascade of ISC pathways of S_1_ → T_1_ → S_0_. Yet, two distinct components, blue and green, are retrieved from these time-resolved, pair-correlated data. We ascribed the blue component to the TS-path, and the green to the one bypassing the TS.

Assigning the major blue-feature (∼76%, accounting for both low and high *j*_CO_ data) to the TS-path may sound provocative in view of the current perception that roaming dominates the acetaldehyde photodissociation with a minor contribution from the TS mechanism.^9–12,16,31^ The key argument for roaming was based on the comparison of the experimental CH_4_ internal energy and *j*_CO_ distributions with the quasiclassical trajectory (QCT) results. A huge discrepancy was found when the direct-dynamics trajectories were initiated at the TS_cc_(S_0_) saddle point, while a closer accordance – albeit notable discrepancies still remained^31^ – was obtained for trajectories starting from the acetaldehyde equilibrium minimum. A similar disagreement between the experiment and an estimated QCT-TS was found in this work on the CH_4_ internal energy distribution (Fig. S7†).

On the other hand, assigning the dominant blue-component as the result of the CH_3_⋯HCO roaming reaction appears at odds with such high *j*-states of CO(*j* ∼ 43) products and large recoil energy (∼29 kcal mol^−1^ or *f*_TKER_ ∼ 0.30). Particularly worth noting from Fig. 3 is the striking variation of the relative intensities of the three partitioned components with the probed *j*_CO_-states, which is obvious for the lower energy (red/green) components, but not so for the high energy (blue) feature. Recall that the correlated distribution of two concomitant products is the key experimental hallmark to differentiate roaming from the TS mechanism in the H_2_CO benchmark.^5,6^

Then, how do we reconcile the dilemma? As mentioned earlier and clearly demonstrated in Fig. 3, upon photolysis of CH_3_CHO at 267 nm all CH_4_ products are born extremely hot, and a majority of them possess internal energy contents very close to the C–H dissociation limit. Due to the multi-reference nature, the CCSD(T) or any other single-reference based methods may fail to provide an accurate description of the dynamical properties of such highly excited CH_4_ products.^32^ Yet, almost all the QCT dynamical simulations^9,10,16,31,33^ have been performed on the basis of the CCSD(T) calculated S_0_-PES. It is also known that the QCT results suffer from zero-point energy leakage,^34,35^ where a part of the energy of the high-frequency modes is transferred to the low-frequency ones, thus resulting in an unrealistic energy distribution. Obviously, this deficiency becomes more problematic for acetaldehyde than formaldehyde.

In addition, a number of new pathways may open up and produce CO + CH_4_ upon photolysis at 267 nm. Previous *ab initio* calculations,^36^ also confirmed in this study, indicated three TS structures for the C–C bond cleavage on the S_0_ surface. The lowest energy one, as shown in Fig. 3, with a C–H–C three-center structure has been assumed to be the only TS_cc_(S_0_) in all previous QCT calculations. Nonetheless, the presence of multiple transition states and presumably the associated valley–ridge inflection regions^37,38^ underscore the complexity of this multi-dimensional PES and the challenge in accurate dynamical simulations. At present, as to the origin of the discrepancy between the experimental assignment and the QCT-TS result, it is not yet totally clear. Further theoretical investigations are warranted.

The rate of formation of the green component in the CO + CH_4_ channel is comparable to that of the blue component of the same product channel, which differs from all other pathways (Table 1). Its yield is sensitive to the probed *j*_CO_ state (Fig. 3) – similar to the roaming-mediated red component, yet with a significantly larger TKER than the latter (*f*_TKER_ = 0.13 *versus* 0.05). These distinct characteristics (or properties) of the green component strongly suggest a different pathway from the red and blue paths. On intuitive ground, we posit that the formation of this green component (path ⑤) could be the result of the bifurcation of the trajectories from the valley–ridge inflection point on the S_0_ surface – a dynamically driven pathway or non-TS mechanism.^2,37–39^

## 6. Implications and outlook

As clearly elucidated in the formaldehyde case, a roaming event arises from the self-reaction of a frustrated radical dissociation at a long distance, resulting in an anomalous product pair-correlated distribution. The undisputable roaming signature for acetaldehyde should, by analogy, be manifested in the correlated behavior of CO(*j*) + CH_4_(*v*). As demonstrated here by the time- and state-resolved *P*(*E*) measurements, the two-dimensional view – time and pair-correlation – is particularly illuminating to unravel the complexities and permits new physical insight that is otherwise inaccessible by merely probing the final product distributions. Because of the high dimensionality of a polyatomic molecule, numerous isomeric structures are ubiquitous in the (conceivably rugged) potential landscape of a typical medium-to-large molecule. Many of them, despite being situated at higher internal energies, can readily be accessible upon visible/UV excitation. We surmise that isomerization of energized molecules prior to dissociation might well be the rule rather than the exception in many polyatomic unimolecular processes.

One may address a deeper and more intriguing question: “what are the deciding factors that guide a trajectory towards the roaming region or to follow the tight TS path”? We conjecture that the answer might be traced back to how the system behaves in the vicinity of a TS: the response to the topographical energy landscape (*e.g.*, the presence of a valley–ridge inflection point or a second-order saddle point^40^) and/or the dynamical behaviors of a highly energized molecule as it traverses the barrier.^39^ Some trajectories, instead of climbing over the barrier through a narrow window, may stray far from the MEP and skirt around the barrier by the entropic advantage of diverse, alternative paths because of the rugged, high-dimensional PES. As inferred above for path ⑤, those non-TS trajectories on S_0_ experience very different forces and could, in principle, proceed further by a dynamically driven migration of the aldehyde-H atom to produce CH_4_ + CO, leaving distinct non-MEP imprints in product attributes. Hence, the barrier-skirting route may or may not lead to a roaming event, which usually refers to an orbiting self-reaction of failed radical dissociation near the asymptote. In other words, roaming seems to require both non-MEP paths at a short range and orbiting radical-recombination at a long distance in a flat region of the PES.

Clearly, a conceptual framework of the roaming phenomenon is far from complete: one needs to comprehend not only the dynamical description near the asymptote, which has been amply investigated theoretically, but also how and why the trajectories get there. A promising theoretical approach based on the geodesic paths (not classical trajectories) has recently been formulated and applied to the formaldehyde benchmark.^41^ That study scrutinized the onset of the paths that will eventually roam and suggested that their stochastic character in the early stage sets the destination. A nonlinear dynamics theory from the phase space perspective, albeit with reduced dimensionality, has also been developed to frame and to analyze the roaming phenomenon and dynamical characteristics.^42^ Extending these new approaches to acetaldehyde can not only further sharpen our understanding of the nature of the non-MEP paths and roaming events proposed here, but can also pave the road for systems of higher dimensions in general.

## Methods

### Experiment

The experiment employed the typical pump–probe spectroscopic technique with a picosecond (ps, 10^−12^ s) resolution [see Fig. S1† for the experimental setup]. A 267 nm laser pulse initiated the photochemical process by exciting CH_3_CHO to the S_1_ state. The probe laser, using a (2 + 1) REMPI scheme *via* the 3p_*z*_ transition at 333 nm to probe the CH_3_ radical or *via* the B-state at ∼230 nm for either the low-*j* (*j* ∼ 0) or the high-*j* (*j* ∼ 43 at peak) of CO(*v* = 0) (Fig. S2†), was fired at variable delays to track the formation of the targeted products. Both pump and probe lasers were derived from a kHz 100 fs (femtosecond, 10^−15^ s) laser system, modified to ps in this study with a pulse duration of ∼1.7 ps and a ∼2 times transform-limited frequency bandwidth. The reactant CH_3_CHO was delivered to a vacuum chamber from a pulsed molecular beam running at 500 Hz. Two modes of data acquisition were adopted: the time-of-flight (TOF) mass spectrometry registered the temporal evolution of all correlated fragments and the time-sliced, velocity-mapped image^43–45^ for a given product at a preset pump–probe delay informed the time-dependent, pair-correlated state distribution.

### Partitioning the time-dependent *P*(*u*; Δ*t*) distributions

In order to decompose the entangled multicomponent contributions from the observed, time-dependent *P*(*u*; Δ*t*) distributions in a least unbiased manner, we posited that each component will correspond to a single reaction pathway, which can be characterized by a unique rate of formation and/or the distinct product pair-correlated distribution. With this assumption, in conjunction with the TOF-normalized image results, we partitioned each time-dependent *P*(*u*; Δ*t*) distribution at a given Δ*t* into 20 or more segments in the velocity space and then examined the temporal evolution of each segment. We found that the profiles in the fast and slow recoil velocity regions can indeed be respectively fitted by a single exponential growth profile with unique time constants. For the intermediate or overlapped speed region, a double- or triple-exponential growth form was invoked, and their relative magnitudes were varied in the fit under the constraint of a consistent growth rate for each individual component (or reaction pathway).

### Theory

The stationary and intersection structures, which are relevant to the radical (CH_3_ + HCO) and molecular (CH_4_ + CO) channels that occur on the S_0_, T_1_, and S_1_ surfaces of CH_3_CHO, were firstly optimized by using a three-state averaged complete active space self-consistent field method (SA3-CASSCF), together with the 6-311G** basis set. The optimized structures were confirmed to be minimum-energy or first-order saddle points by harmonic frequency calculations. The active space for the SA3-CASSCF calculation is composed of 14 electrons in 13 orbitals, referred to as SA3-CAS(14,13), which include all the valence orbitals of CH_3_CHO, except for the σ and σ* orbitals of the C–O bond. In order to account for dynamical electron correlation, the single-point energies were calculated with multi-state second-order perturbation theory on the basis of the SA3-CAS(14,13)/6-311G** optimized structures and calculated electronic wave functions, referred to as MS-SA3-CASPT2(14,13)/6-311G**. Further calculations were performed for the S_0_ and T_1_ states with the B3LYP functional and MP2 methods, along with the cc-pVTZ basis set. The B3LYP/cc-pVTZ and MP2/cc-pVTZ calculated results are well consistent with those from the MS-SA3-CASPT2(14,13)/6-311G** calculations, which justifies the reliability of the present theoretical calculation. In comparison with the experimental findings where available, the B3LYP/cc-pVTZ calculations provide a slightly better estimation for the S_0_ and T_1_ states. Therefore, the relative energies shown in Fig. S8† come from the MS-SA3-CASPT2(14,13)/6-311G** calculation for the S_1_ state and the B3LYP/cc-pVTZ calculation for the S_0_ and T_1_ states.

## Data availability

The data that support the findings of this study are available from the corresponding author upon request.

## Conflicts of interest

The authors declare no competing financial interests.

## Supplementary Material

SC-011-D0SC00063A-s001

SC-011-D0SC00063A-s002
