# Supplementary material for: Real-time tracking of the entangled pathways in the multichannel photodissociation of acetaldehyde
Source: Chem Sci. 2020 Feb 26;11(25):6423–30. doi: 10.1039/d0sc00063a (PMC8159351; doi:10.1039/d0sc00063a)
Supplement: SC-011-D0SC00063A-s001 [file SC-011-D0SC00063A-s001.pdf]

Electronic Supplementary Material (ESI) for Chemical Science.
This journal is © The Royal Society of Chemistry 2020

Online Supplementary Materials for

Real-time tracking of the entangled pathways in the multichannel photodissociation of acetaldehyde

Chung-Hsin Yang,[1]† Surjendu Bhattacharyya,[1]† Lihong Liu,[2]† Wei-hai Fang,[2]* Kopin Liu[1,3,4]*

[1] Institute of Atomic and Molecular Sciences (IAMS), Academia Sinica, P. O. Box 23-166, Taipei, Taiwan 10617.

[2] Key Laboratory of Theoretical and Computational Photochemistry, Ministry of Education, Department of Chemistry, Beijing Normal University, Beijing 100875, P. R. China.

[3] State Key Laboratory of Molecular Reaction Dynamics, Dalian Institute of Chemical Physics, CAS, Dalian 116023, P. R. China.

[4] Aerosol Science Research Center, National Sun Yat-sen University, Kaohsiung, Taiwan 80424.

† These authors contributed equally to this work.

* To whom correspondence should be addressed. Email: fangwh@bnu.edu.cn (W.F.); kliu@po.iams.sinica.edu.tw (K.L.)

This PDF file includes:

Figs. S1 to S8

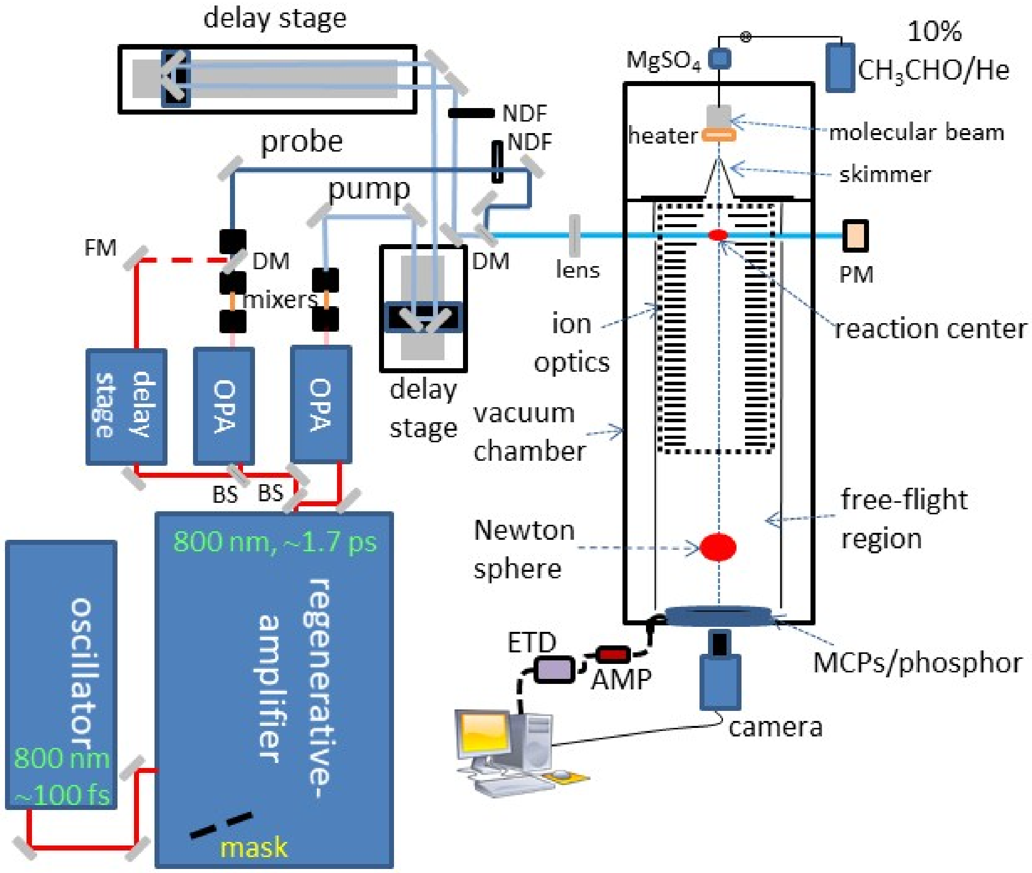

Fig. S1 Schematic of experimental setup. Abbreviation: BS: beam splitter; OPA: optical parametric amplifier; DM: dichroic mirror; FM: flip mirror; NDF: neutral-density filter; PM: power meter; MCPs: microchannel plates; ETD: event time digitizer; AMP: preamplifier.

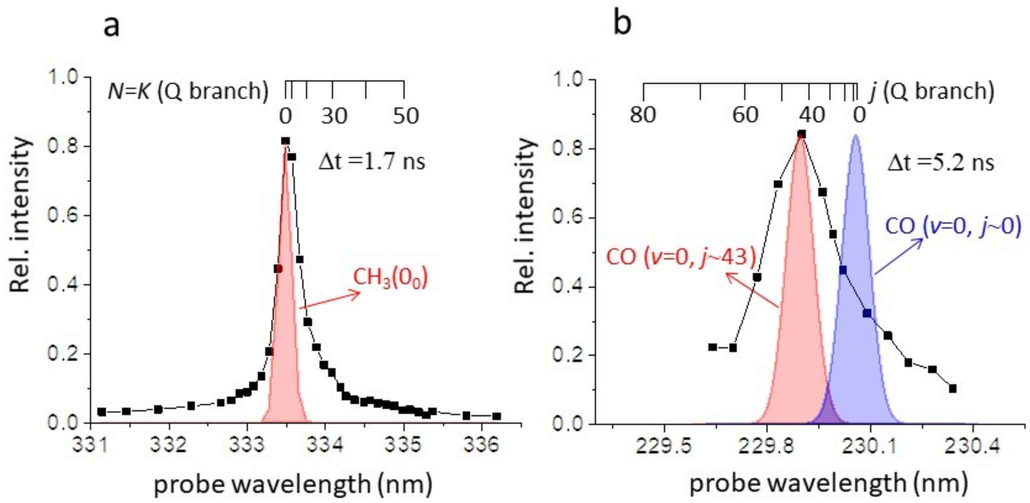

Fig. S2 REMPI spectra of the probed products. **(a)** (2+1)-REMPI spectrum for the $CH_3(3p_z\ ^2A_2$" $(v'= 0) \leftarrow X\ ^2A_2$" $(v''= 0)$) and **(b)** the CO($B\ ^1\Sigma^+(v'= 0) \leftarrow X\ ^1\Sigma^+(v''= 0)$) transitions. The black dots are the experimental measurements, while the color-filled curves represent the range of the probe wavelengths for three products, assuming a Gaussian profile with a ~18 cm^{-1} linewidth (FWHM). Note the nonzero baselines of the spectra, which arise from dissociative ionization backgrounds displaying an exponential decay behavior with the same time constant of 190 ps for both products.

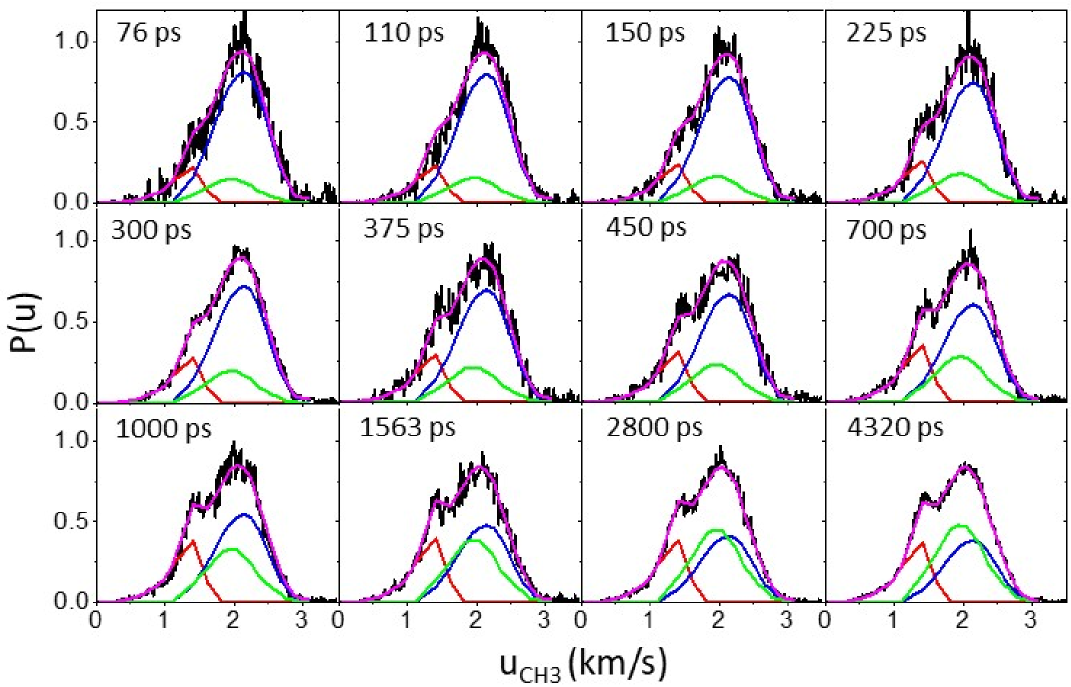

Fig. S3 P(u; Δt) distributions of $CH_3(0_0)$ at different pump-probe time delays. The color code is the same as that in Fig. 2c.

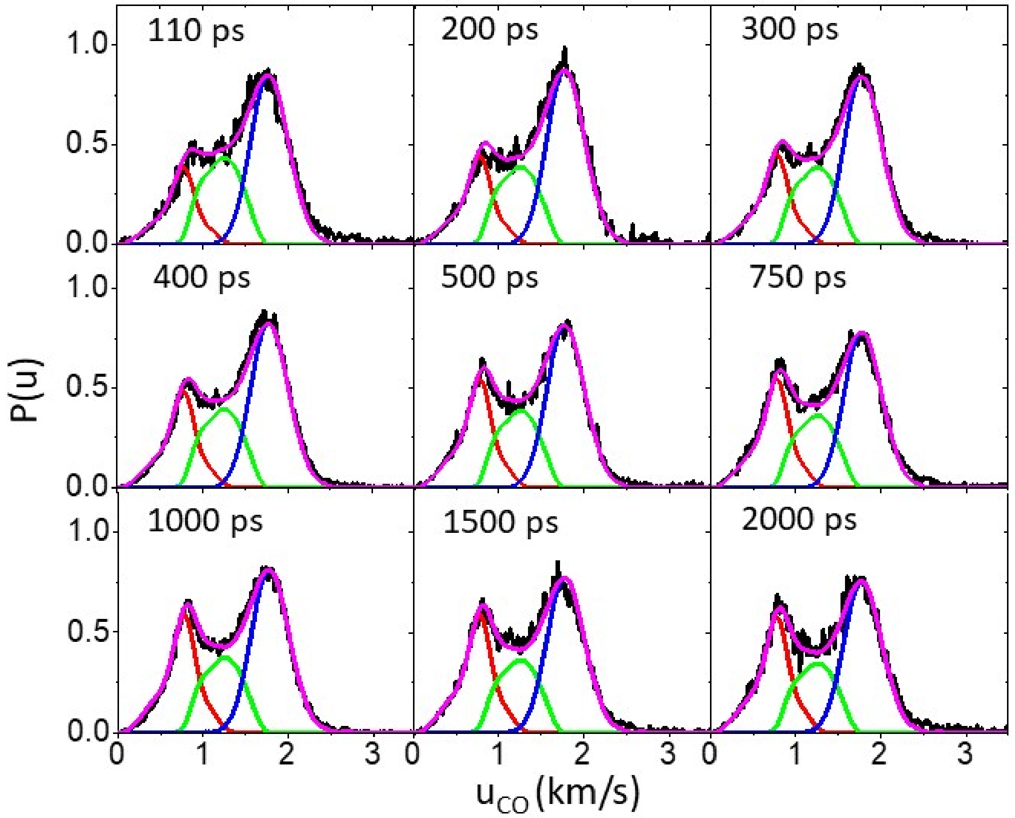

Fig. S4 P(u; Δt) distributions of CO (v = 0, j ~ 0) at different pump-probe time delays. The color code is the same as that in Fig. 2c.

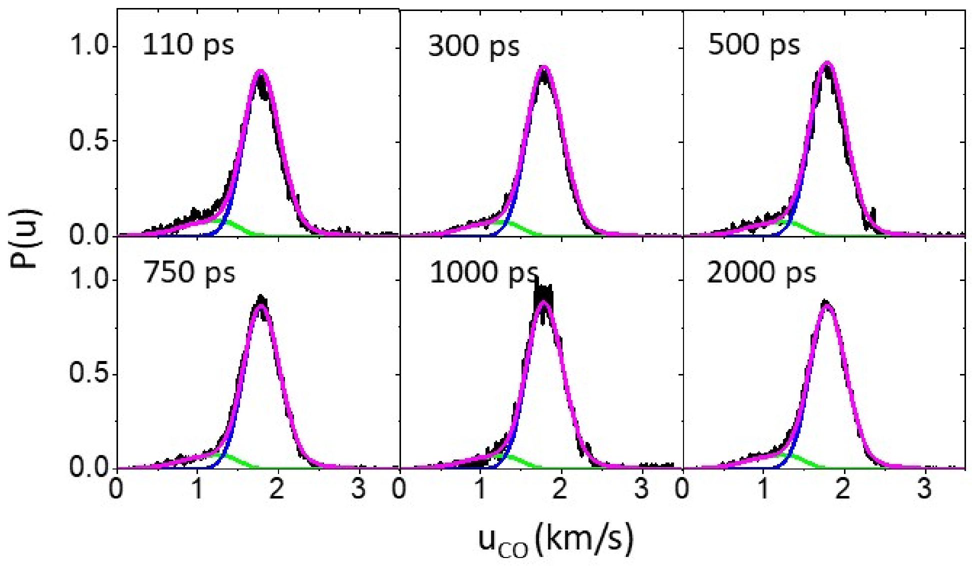

Fig. S5 P(u; Δt) distributions of CO (v = 0, j ~ 43) at different pump-probe time delays. The color code is the same as that in Fig. 2c.

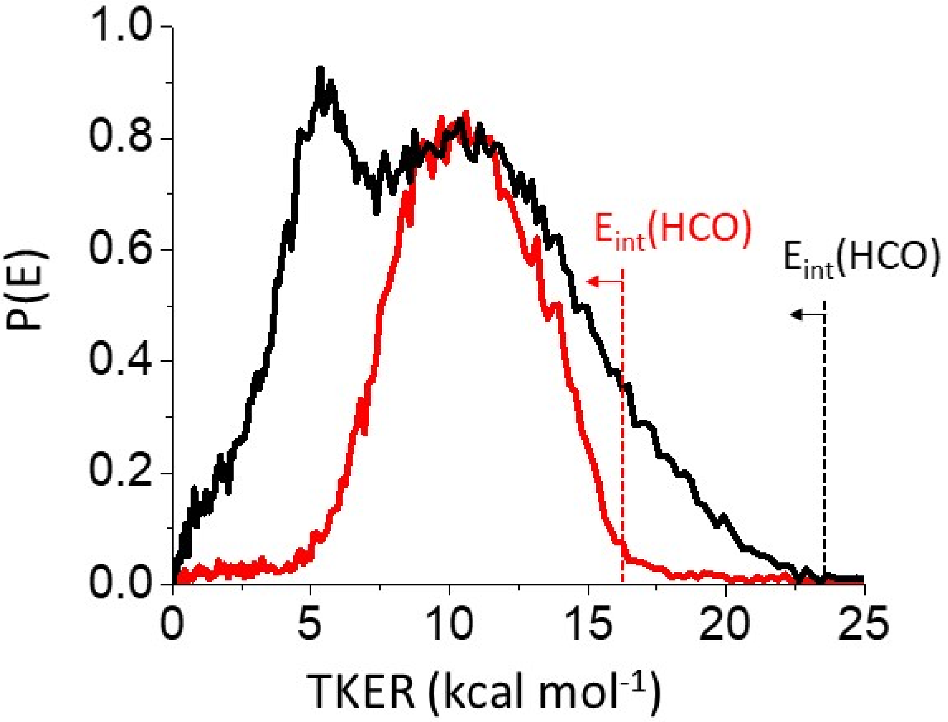

Fig. S6 Effects of different photolysis wavelengths. P(E) distributions of $CH_3(0_0)$ + HCO from photolysis at 266.7 nm (black) and at 286.0 nm (red).

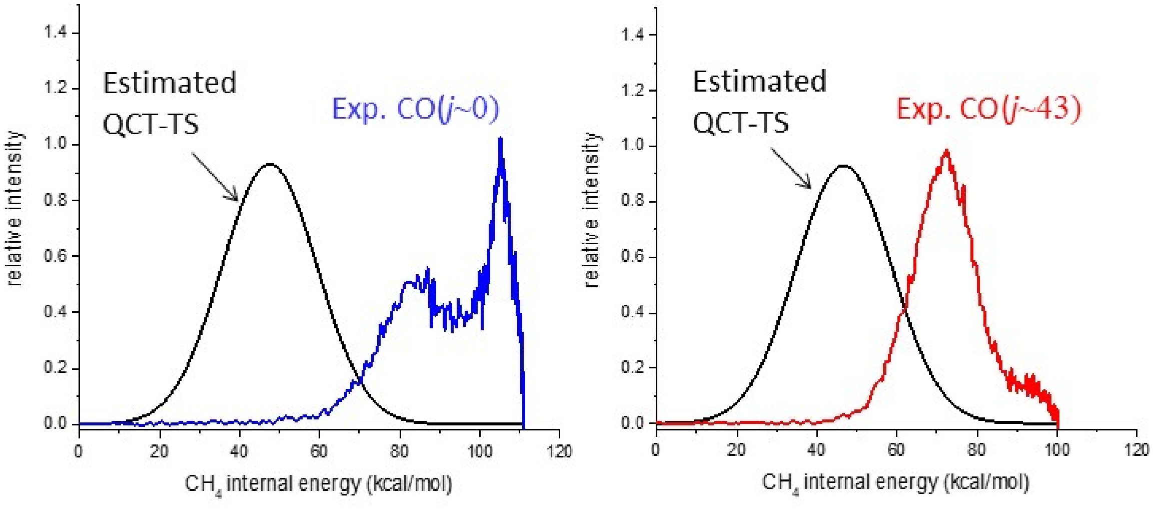

Fig. S7 Experiment and theory comparison. Comparison of experimental P(E) distributions of the CO + CH_4 channel with QCT results that are based on direct dynamics calculation initiated from the saddle-point TS on the S_0 surface. The QCT-TS shown here is estimated from the calculations for photolysis at 308 nm[9,10,31] and 248 nm[16,33] in the literature.

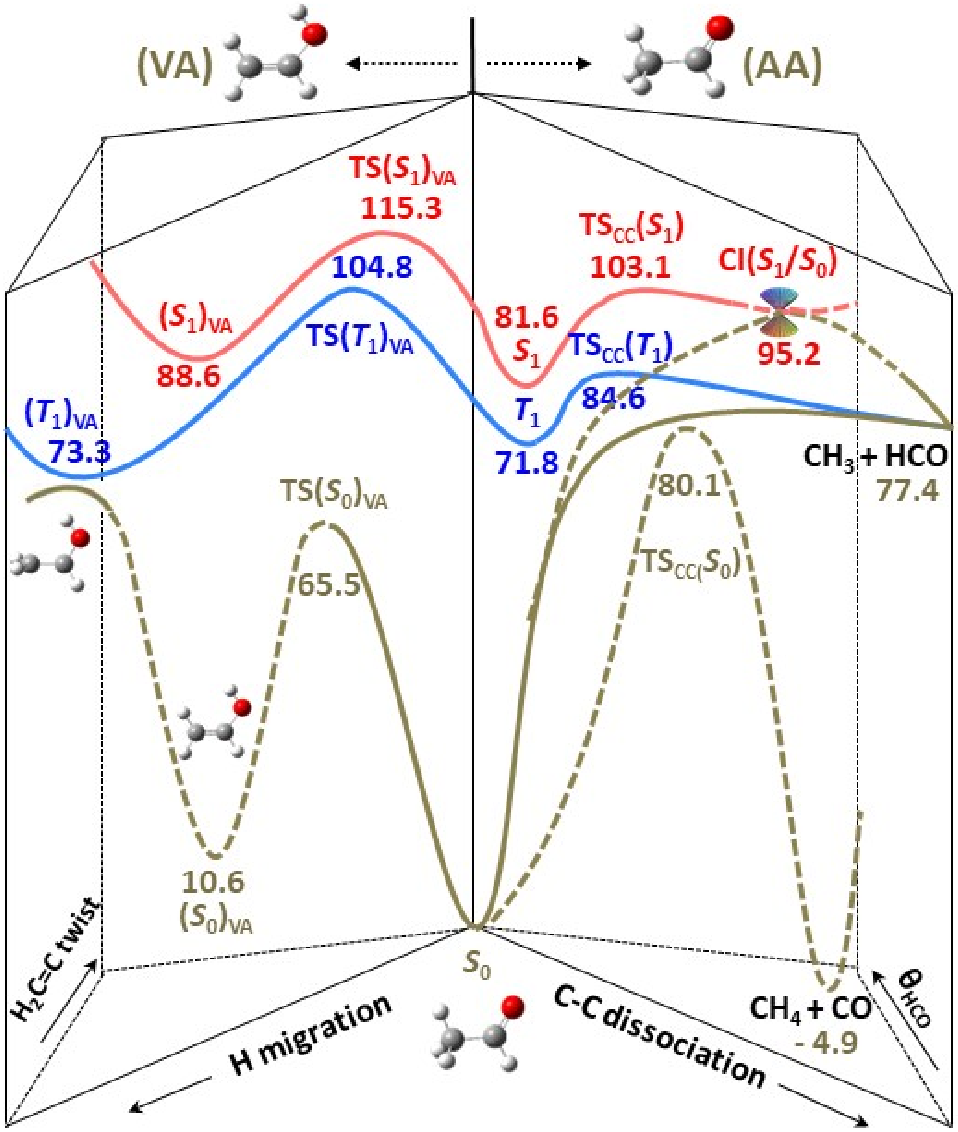

Fig. S8 Potential energy profiles for the CH_3CHO (AA) decomposition into CH_3 + HCO and CH_4 + CO, as well as isomerization to CH_2CHOH(VA) on the S_0, T_1, and S_1 states. The relative energies (kcal mol^{-1}) of the stationary and intersection structures are from the MS-SA3-CASPT2(14,13)/6-311G** calculation for the S_1 state and the B3LYP/cc-pVTZ calculation for the S_0 and T_1 states.
