# Supplementary material for: Real-time tracking of the entangled pathways in the multichannel photodissociation of acetaldehyde
Source: Chem Sci. 2020 Feb 26;11(25):6423–30. doi: 10.1039/d0sc00063a (PMC8159351; doi:10.1039/d0sc00063a)

Electronic Supplementary Material (ESI) for Chemical Science.
This journal is © The Royal Society of Chemistry 2020

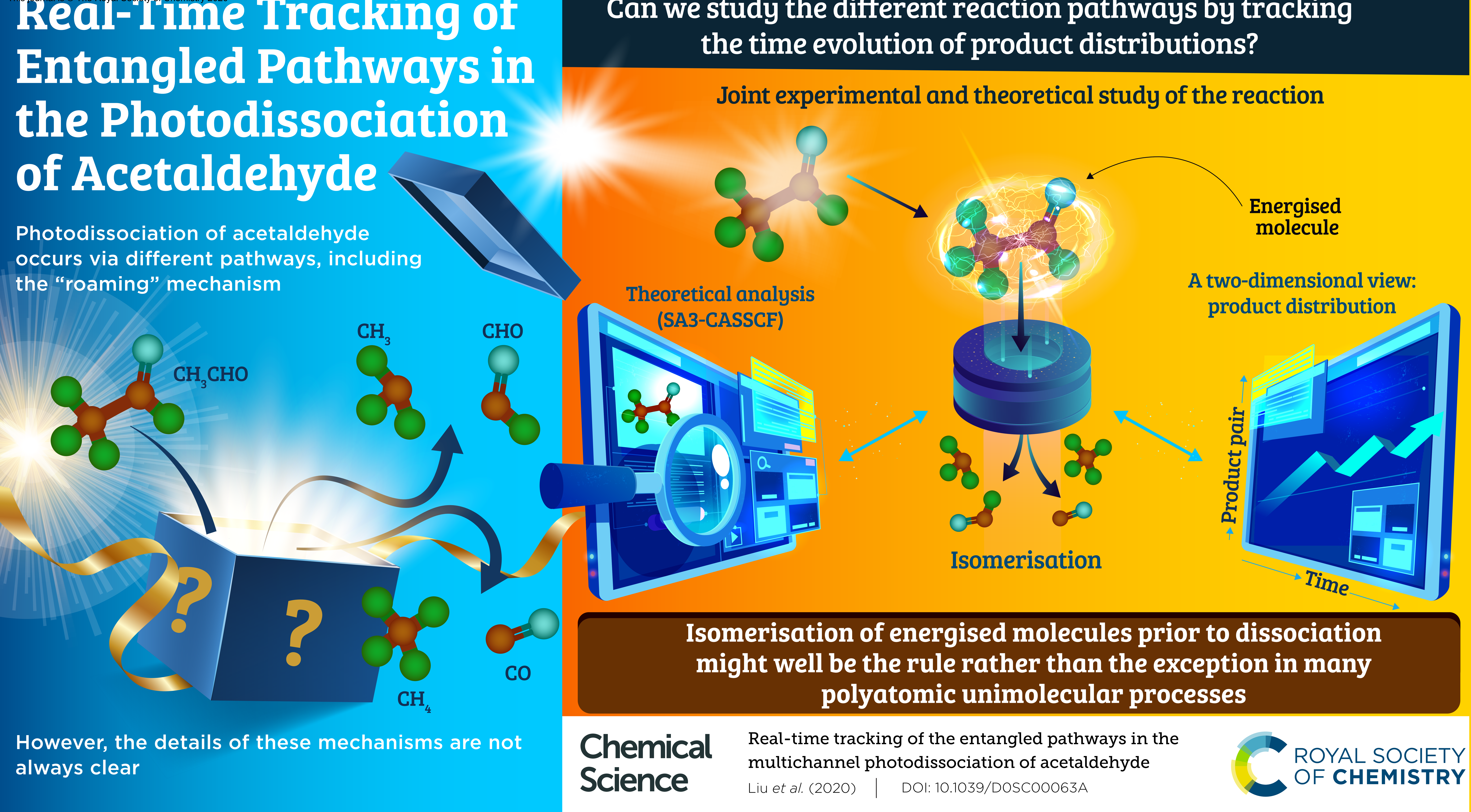

Supplement: SC-011-D0SC00063A-s002 [file SC-011-D0SC00063A-s002.pdf]
